# Supplementary material for: Content-rich biological network constructed by mining PubMed abstracts
Source: BMC Bioinformatics. 2004 Oct 8;5:147. doi: 10.1186/1471-2105-5-147 (PMC528731; doi:10.1186/1471-2105-5-147)
Supplement: Additional File 2 — The original results of the above study (non-essential files are deleted to keep the file size under the limit set by BMC bioinformatics). [file 1471-2105-5-147-S2.bz2 › chilibotAdditionalFile2/dip20/25ID8999548E88/html/CASP1_IL1B.html]

 


 **CASP1** and **IL1B** 
  
Found 435 abstracts in PubMed, retrieved 20.  
 

 What does Google say? 
 PDF only 
| .edu only 

---

**Interactive relationship** (e.g. stimulation, inhibition, etc)

**Neutral relationship**- Amebic lysates have also been shown to activate the precursor to interleukin 1 beta  [ **IL1B** ]  proIL 1beta, mimicking the action of caspase 1  [ **CASP1** ] .  Ref: 12595442 Infect Immun, 2003

**Non-interactive relationship** (e.g. studied together, co-existance, homology, etc.)

- Cells on oxidized matrix demonstrated enhanced expression of Bax, increased fragmentation of PARP, and diminished apoptosis in the presence of the interleukin 1 beta  [ **IL1B** ]  converting enzyme  [ **CASP1** ]  inhibitor Ac Tyr Val Ala Asp aldehyde.  Ref: 11922616 Biochem Biophys Res Commun, 2002
- Co expression of PYPAF7 and ASC results in the synergistic activation of caspase 1  [ **CASP1** ]  and a corresponding increase in secretion of interleukin 1 beta  [ **IL1B** ] .  Ref: 12019269 J Biol Chem, 2002
- In the lining like layers and deep stroma of SMLIT, many cells were double positive for active caspase 1  [ **CASP1** ]  and interleukin 1 beta  [ **IL1B** ]  IL 1beta.  Ref: 12111083 Rheumatol Int, 2002
- Shigella flexneri infection of human macrophages is followed by rapid bacterial escape into the cytosol and secretion of IpaB, which activates caspase 1  [ **CASP1** ]  to mediate cell death and release of mature interleukin IL 1 beta  [ **IL1B** ] .  Ref: 12065527 Infect Immun, 2002
- Using primary cultured murine peritoneal macrophages, we demonstrate that P2X7 receptor activation causes release of IL 1 beta  [ **IL1B** ]  and IL 1 alpha via a common pathway, dependent upon the release of Ca 2 from endoplasmic reticulum stores and caspase 1  [ **CASP1** ]  activity.  Ref: 12626557 J Immunol, 2003
- The LPS induced effects were abrogated by inhibition of caspase 1  [ **CASP1** ] , suggesting that IL 1 beta  [ **IL1B** ]  may mediate the effects of LPS.  Ref: 12609991 J Biol Chem, 2003
- Effect of TNF alpha on the induction of apoptosis in murine macrophages role of interleukin 1 beta  [ **IL1B** ]  converting enzyme  [ **CASP1** ] .  Ref: 12622883 Int J Immunopathol Pharmacol< MedlineTA>Int J Immunopathol Pharmacol, 2003
- These results suggest that lemon pure essential oils may modulate the apoptosis through the activation of the interleukin 1 beta  [ **IL1B** ]  converting enzyme  [ **CASP1** ]  like caspases.  Ref: 12162947 Pharmacol Res, 2002
- As cytoplasmatic adaptor molecules of FAS, e.g. FLIP Fas associated death domain protein FADD like interleukin 1 beta  [ **IL1B** ]  converting enzyme  [ **CASP1** ]  FLICE caspase 8 inhibitory protein, also modulate TRAIL signals, we determined whether chelerythrin affected TRAIL mediated apoptosis.  Ref: 12877678 Br J HaematolBr J Haematol, 2002
- Changes in caspase 3 and caspase 1  [ **CASP1** ] , interleukin 1 beta  [ **IL1B** ]  IL 1 beta  [ **IL1B** ] , and collagen I, III, and IV proteins and mRNA were detected by Western blotting and Northern blotting, respectively.  Ref: 12753292 Kidney Int, 2003
- RESULTS Expression of TNF alpha, IL 1 beta  [ **IL1B** ] , caspase 1  [ **CASP1** ]  and NOS2 was found in the cytoplasm and sarcolemma of dystrophic skeletal muscle fibres.  Ref: 12673888 Clin Exp Rheumatol< MedlineTA>Clin Exp Rheumatol, 2003
- Vertex is collaborating with Aventis Pharma AG formerly Hoechst Marion Roussel Inc in the development of pralnacasan, an interleukin IL 1b  [ **IL1B** ]  converting enzyme ICE  [ **CASP1** ]  inhibitor, for the potential treatment of inflammatory diseases 17024 8829 53094.  Ref: 12789619 Idrugs, 2003
- METHODS Immunohistochemical staining of skeletal muscle biopsies 10 DM, 10 SSc, 10 PM, 10 SLE, 10 non inflammatory myopathies for tumour necrosis factor alpha TNF alpha, interleukin 1 beta  [ **IL1B** ]  IL 1 beta  [ **IL1B** ] , activated caspase 1  [ **CASP1** ] , pan macrophage marker CD68, inducible nitric oxide synthase NOS2 and nerve growth factor receptor NGFR.  Ref: 12673888 Clin Exp Rheumatol< MedlineTA>Clin Exp Rheumatol, 2003
- PURPOSE To detect cell specific apoptosis factors, Fas and Fas ligand, and the common intracellular apoptosis modulators, interleukin 1 beta  [ **IL1B** ]  converting enzyme  [ **CASP1** ]  ICE  [ **CASP1** ]  like protease caspase 1  [ **CASP1** ] , Bcl 2, Bcl xL and Bax in lens epithelial cells LEC of human cataracts.  Ref: 11803481 Curr Eye Res, 2001
- CONCLUSION Prominent expression of the proinflammatory factors TNF alpha, IL 1 beta  [ **IL1B** ]  and NOS2 and caspase 1  [ **CASP1** ]  is associated with muscle fibre damage, albeit when expressed to a low degree these factors may, like NGFR, contribute to muscle regeneration and healing.  Ref: 12673888 Clin Exp Rheumatol< MedlineTA>Clin Exp Rheumatol, 2001
- Dual effects of caspase 1  [ **CASP1** ] , interleukin 1 beta  [ **IL1B** ] , tumour necrosis factor alpha and nerve growth factor receptor in inflammatory myopathies.  Ref: 12673888 Clin Exp Rheumatol< MedlineTA>Clin Exp Rheumatol, 2001
- Of the 4 cDNA fragments, 2 A1 and D1 did not show sequence homology with the existing genes in the GeneBank, one A2 was 89% homologous with interleukin 1 beta  [ **IL1B** ]  converting enzyme  [ **CASP1** ]  ICE  [ **CASP1** ]  gene, and the other D2 was identical to mouse musculus 45s pre rRNA MM45srRNA gene.  Ref: 11783011 Zhonghua Zhong Liu Za Zhi, 2001
- The cellular Fas associated death domain like interleukin 1 beta  [ **IL1B** ]  converting enzyme  [ **CASP1** ]  inhibitory protein long form FLIP L, a structural homologue of caspase 8 lacking caspase activity because of several mutations in the active site, is a potent inhibitor of death receptor induced apoptosis.  Ref: 12215447 J Biol Chem, 2002
